# Long-Term Infusion of Acylated Ghrelin Blunts LH Surge and Diminishes the Superovulatory Response in Dairy Sheep

**DOI:** 10.3390/ani15121767

**Published:** 2025-06-15

**Authors:** Ilias Ramouzis, Leda Oikonomopoulou, Ioannis Nanas, Konstantina Stamperna, Georgios S. Amiridis, Eleni Dovolou

**Affiliations:** 1 Department of Obstetrics & Reproduction, Faculty of Veterinary Science, University of Thessaly, 43100 Karditsa, Greece; iliasramouzis@gmail.com (I.R.); l_oikonomopoulou@yahoo.gr (L.O.); gnsnanas@gmail.com (I.N.); konstantina.stamperna@gmail.com (K.S.); 2 Laboratory of Reproduction, Department of Animal Sciences, University of Thessaly, 41334 Larissa, Greece; entovolou@uth.gr

**Keywords:** ghrelin, LH, progesterone, anti-mullerian hormone, superovulation, embryo, sheep

## Abstract

Ghrelin, the endogenous ligand of the growth hormone secretagogue receptor, is released pre-prandially and during periods of negative energy balance, exhibiting anti-fertility properties. In this study, twenty ewes were divided into two groups: a ghrelin-treated group receiving 1.25 μg/kg body weight (BW) of ghrelin per day via mini-pumps for 28 days and an untreated control group. Estrus was synchronized, superovulation was induced with FSH, and embryos and follicular fluid were collected six days post-estrus. Blood samples were taken to measure LH, progesterone, and anti-Müllerian hormone (AMH) concentrations. Results indicated that in treated animals, preovulatory LH surge was weaker, and progesterone levels were lower than in controls. Differences were observed in the superovulatory response and the number of collected embryos, both being higher in controls. While AMH levels did not differ between groups at the beginning of the experiment, they were lower in treated animals at the time of FSH administration. Treated ewes exhibited a reduced number of small follicles, and their follicular fluid contained lower AMH concentrations than the controls. These findings suggest that ghrelin plays a direct role in regulating LH secretion from the pituitary and in controlling ovarian follicle development, highlighting the strong interaction between nutrition and fertility.

## Simple Summary

Negative energy balance is a significant factor that suppresses fertility in nearly all mammals. Ghrelin, a potent appetite-stimulating hormone, is primarily secreted from the stomach before meals and during periods of reduced energy availability. It is known to inhibit various reproductive functions as a protective mechanism for the mother and fetus against energy deficiency. In this study, we utilized a sheep model to investigate the effects of prolonged exposure to ghrelin on the pituitary gland and ovaries. Twenty ewes were fitted with mini pumps to deliver continuously for 28 days ghrelin or saline. Superovulation was hormonally induced, and various reproductive hormones were assayed. Our findings demonstrated that ghrelin decreased luteinizing hormone (LH) levels, which are essential for ovulation, as well as progesterone concentrations. Furthermore, follicular development was impaired, evidenced by a reduced number of developing follicles. Additionally, ovarian follicles did not respond adequately to exogenous hormones that typically induce multiple follicle development. These results provide strong evidence that ghrelin, in addition to its known suppressive effects on the hypothalamus and pituitary gland, interferes with follicular development, potentially altering the functions of follicular cell populations.

## 1. Introduction

In female mammals, particularly dairy ruminants, maintaining high fertility is a highly energy-demanding process, as conception and fetal development usually coincide with lactation. Consequently, reproductive performance is closely linked to metabolic status. Both functions are regulated by a complex neuroendocrine network in the brain, involving interactions among metabolic signals, hormones, hormonal mediators, and neuropeptides [1,2,3]. During periods of prolonged negative energy balance (NEB), dysregulation of the hypothalamus–pituitary–ovarian (HPO) axis occurs, characterized by increased blood growth hormone (GH) concentrations and reduced glucose and insulin levels. These alterations can lead to disrupted ovulation or anestrus [4,5,6].

Ghrelin, a polypeptide hormone discovered in 1999, is recognized as a potent appetite stimulant secreted prior to meal consumption [7]. In sheep, elevated ghrelin concentrations are observed before scheduled feeding and remain high during periods of reduced energy supply [8,9]. Ghrelin is primarily secreted by X/A oxyntic cells in the stomach [7,10], but its expression and that of its receptor have also been identified in the brain, intestine, liver, kidneys, pancreas, and ovaries [11]. The hormone circulates in two forms: the acylated form, which has an n-octanoyl group attached to the serine 3 residue, and the non-acylated form [7]. Ghrelin’s role in promoting GH secretion, stimulating food intake, and influencing energy balance and body weight has been well-documented [12,13]. Increased feed intake and appetite stimulation result from interactions among various factors, including environmental influences, hormones, metabolites, and neuropeptides [14,15]. The balance between two neuronal populations in the arcuate nucleus of the hypothalamus—the anorexigenic pro-opiomelanocortin (POMC) neurons and the orexigenic neuropeptide Y (NPY)/agouti-related peptide (AgRP) neurons—serves as a major regulator of feed intake [16]. Ghrelin stimulates appetite by inhibiting POMC neurons and activating NPY neurons, leading to orexin release from the lateral hypothalamic area [17,18,19,20]. Ghrelin also plays a crucial role in glucose metabolism through the modulation of insulin secretion; however, its effects depend on the nutritional status of the animals. In fasting sheep, ghrelin inhibits glucose-induced insulin secretion, while it stimulates insulin release in well-fed animals [21]. Additionally, environmental factors related to metabolism and pregnancy status have been shown to influence circulating ghrelin levels in cattle [22,23]. Long-term infusion of des-acyl ghrelin (a truncated form) has been associated with body condition score loss and reduced concentrations of IGF-1, glucose, and leptin, alongside increased non-esterified fatty acid (NEFA) levels—conditions resembling NEB exposure [24,25].

Beyond its established role in metabolism, ghrelin has emerged as a significant regulator of reproduction at both central and peripheral levels. Administration of acylated ghrelin, whether centrally or peripherally, inhibits luteinizing hormone (LH) secretion across multiple species [26,27,28] and attenuates GnRH-induced preovulatory LH and FSH surges in sheep and cattle, indicating a suppressive role at the pituitary level [27,28]. In vitro studies have demonstrated that ghrelin disrupts normal oocyte maturation and decreases blastocyst formation rates by altering gene expression related to oxidation, metabolism, and apoptosis in oocytes and cumulus cells [29,30].

Moreover, ghrelin affects the expression of key steroidogenic enzymes such as HSD3B, hydroxysteroid 17-beta dehydrogenase 1 (HSD17B1), and cytochrome P450 family 19 subfamily A member 1 (CYP19A1/P450AROM), leading to decreased synthesis of steroid hormones including testosterone, estradiol, and progesterone [31,32].

Given that NEB is a known restrictive factor for fertility and that ghrelin upregulation is associated with NEB, the working hypothesis of this study is that in sheep, ghrelin exerts a suppressive role not only on the hypothalamus and pituitary but also within ovarian compartments. To evaluate this hypothesis, we assessed the characteristics of the GnRH-induced preovulatory LH surge and examined follicular populations and their responses to a superovulatory regimen during long-term continuous ghrelin infusion under controlled nutritional conditions in dairy sheep.

## 2. Materials and Methods

All experimental procedures were conducted in accordance with European standards for animal welfare and were approved by the ethical committee of the Greek Ministry of Rural Development and Food (license number 16033, 1 August 2019).

### 2.1. Animals

Twenty crossbreed ewes [(Karagouniko × Lacaune) × Asaf], aged 291.8 ± 9.2 days with a mean body weight of 43.4 ± 1.2 kg, were utilized during the breeding season. Each group (treated and control, n = 10) was acclimatized in individual pens one week prior to experimentation to facilitate familiarization with the provided diet. All animals were fed twice daily (08:00 and 20:00) with a consistent diet comprising 1.1 kg of alfalfa hay, 0.25 kg of wheat straw, and 0.6 kg of concentrates (cereals, wheat brans, soybean meal, and vitamin and minerals premix) with ad libitum access to drinking water. The diet was adjusted according to recommendations for local and imported sheep breeds [33] recommendations and provided 1.6 kg to 1.6 kg of daily dry matter intake, 1.1 kg of total digestive nutrients (TDN), 0.25 kg of crude protein, 6.3 gr of calcium, and 3.1 gr of phosphorus.

### 2.2. Experimental Design

The experiment was conducted during the breeding season. All ewes were fitted with osmotic mini-pumps capable of delivering their contents for 28 days. The pumps were filled with normal saline (control group C) or with a truncated form of acylated ghrelin (DAP-ghrelin) at a dosage of 1.25 μg/kg/day (treated group T). The dose of DAP-ghrelin was adjusted according to previous findings in sheep [8] and cattle [24]. Ten days post-pump insertion, estrous cycles were synchronized using intravaginal progestogen sponges (60 mg medroxyprogesterone, Ovigest, Hipra SA, Girona, Spain), which remained in situ for 7 days. Superovulation was induced starting 48 h prior to sponge removal, utilizing six decreasing doses of porcine FSH (first, second, and third day, 80 mg/day, 40 mg/day, and 13 mg/day, in total 133 mg/ewe, Folltropin, Vetoquinol SA, Lure Cedex, France), administered every 12 h. A luteolytic prostaglandin F2α injection (125 mcg cloprostenol, Estrumate, MERC, Boxmeer, The Netherlands) was administered along with the 5th FSH dose. Twenty-four hours after sponge withdrawal, a GnRH analog (4 μg buserelin, Receptal, MSD, Boxmer, The Netherlands) was administered, and the ewes were naturally mated to fertile rams. The time of estrus expression–mating (day 0) was recorded for each animal, and uterine flushing for embryo collection was performed six days post-estrus/mating via laparotomy. Following uterine flushing, ovaries were exteriorized, and the corpora lutea and all visible follicles were counted; follicles were aspirated, and separate pools of follicular fluid collected either from small (2–3 mm diameter) and large (>3 mm diameter) from each animal, were stored at −20 °C for AMH assessment. Upon completion of embryo and follicular fluid collection, prostaglandin F2α (125 mcg cloprostenol, Estrumate, MERC, Boxmeer, The Netherlands) was administered. Subsequently, estrus behavior was monitored for three days using apron-fitted rams, and blood samples were collected every other day during the subsequent new cycle for progesterone concentration determination. At the end of the estrous cycle, the ewes were mated to fertile rams, and the interestrus interval was recorded.

### 2.3. Ghrelin Infusion

The hormone used for infusion in treated animals was Dap3-ghrelin (H-Gly-Se-Dap(n-octanoyl)-Phe-Leu_NH2), purchased from Peptides International Inc. (Louisville, KY, USA; catalog number PGH-36881-PI). This truncated acylated peptide has a molecular weight of 633.79 g/mol, facilitating continuous release from the pump over extended periods. The lyophilized Dap-ghrelin was dissolved in 50% *w*/*w* dimethylsulfoxide (DMSO; Sigma Chemical Company, Poole, UK) at a concentration of 1 mg in 125 mL of DMSO. In treated animals, the hormone was delivered via osmotic mini-pumps (ALZET 2 mL 4, ALZET LLC, Campbel, CA, USA), which released 2.5 μL/h over 28 days. The pumps inserted to control animals were filled with saline (0.9% NaCl). Prior to filling, pumps were weighed; the solution was injected using a blunt 25-gauge needle attached to a syringe while avoiding air insertion. The filled pumps were re-weighed and primed overnight in normal saline at 37 °C.

The pumps were implanted midway on the right side of each animal’s neck. The implantation area was shaved and disinfected with povidone-iodine and alcohol; local anesthetic (xylocaine) was administered subcutaneously. A skin incision was made to create a small subcutaneous pocket using hemostat jaws. The filled pump was inserted into the pocket, and the incision was closed with non-absorbable sutures.

### 2.4. Blood Sampling

Blood samples were collected from six animals per group at pump insertion and during the first and last FSH injections for AMH determination. An additional set of ten blood samples was obtained through an indwelling jugular vein catheter within three hours post-GnRH administration for LH concentration analysis at time points −30, 0, 30, 45, 60, 75, 90, 105, 120, and 180 min relative to GnRH administration (time point 0). After sample collection, the catheter was flushed with heparinized saline. Progesterone concentrations were analyzed in blood samples from the same six animals on days 1, 3, 5, 7, 9, 12, and 15 post-estrus (day 0) induced by the prostaglandin administration.

All samples were allowed to clot at room temperature; serum was separated by centrifugation at 3000× *g* for 15 min and stored at −20 °C until hormone analyses.

### 2.5. Embryo Collection

Prior to embryo collection, animals were deprived of feed and water for 18 and 10 h, respectively.

### 2.6. Anesthesia and Embryo Collection

Epidural anesthesia was achieved by injecting a 2% xylocaine solution (0.6 mg/kg BW; Xylosan 2%, DEMO SA, Athens, Greece) into the lumbosacral space to desensitize the abdominal wall caudal to the umbilicus and inguinal region. Local anesthesia was also administered at the sites of trocar insertion. Ovarian response evaluation techniques—counting the corpora lutea—and embryo collection have been previously described [34,35]. Briefly, ovaries were observed endoscopically; embryo collection was carried out only in animals exhibiting moderate (3 to 5 CLs) or good (>5 CLs) ovarian responses. The uterus was exteriorized through a small abdominal incision, and uterine horns were flushed with 80 mL of ready-to-use commercial embryo collection medium containing bovine serum albumin (BSA) and antibiotics (Boviflush, Minitube Int., Tiefenbach, Germany). The flushing medium was injected through a fine needle from the tip of the uterine horn and collected via a two-way Foley catheter (Nanchang Kaimed Medical, Nanchang, China) inserted cranially into the uterine bifurcation. The flushing medium was filtered to isolate embryos under stereomicroscopic observation. The embryos were kept in holding medium containing glucose, pyruvate, BSA, amino acids, growth factors, and antibiotics (BoviHold, Minitube Int., Tiefenbach, Germany).

For follicular fluid collection, ovaries were exteriorized; visible follicles (>2 mm) were counted and aspirated using a 21-gauge needle connected to a 10 mL syringe. Follicular fluid from small (2–3 mm diameter) and large (>3 mm diameter) follicles were pooled separately and stored at −20 °C for AMH concentration determination.

### 2.7. Hormonal Assays

Serum concentrations of LH, progesterone, and AMH were measured using enzyme immunoassay (ELISA) commercial reagent kits according to the manufacturers’ instructions (LH: Cusabio, Wuhan, China, CSB-E12826B; progesterone: DRG, Marburg, Germany, EIA1561; AMH: Mybiosource, San Diego, CA USA, MBS1602135). The detection ranges were 1.25 mIU/mL to 100 IU/mL for LH, 0.08 ng/mL to 50.0 ng/mL for progesterone, and 0.05–15 ng/mL for AMH. The intra-assay and inter-assay coefficients of variation were 8.6% and 9.8% for LH, 6.8% and 5.6% for progesterone, and 7.2% and 9.1% for AMH. Absorbance (optical density) was measured at 450 nm using an automated microtiter plate reader (MRC, Scientific Instruments, Timrat, Israel). All assays were conducted in duplicates, and the mean values were calculated. Samples with a difference greater than 5% between duplicates were re-assayed.

### 2.8. Statistical Analysis

Statistical analyses were performed using IBM SPSS Statistics 25.0 for Windows. Results are expressed as means ± standard deviations. Data were assessed for normal distribution using the Shapiro–Wilk normality test and for homogeneity of variance using Levene’s test. For independent continuous data, Student’s *t*-test and Welch’s *t*-test were utilized as appropriate. One-way repeated measures ANOVA or paired samples *t*-test was employed to evaluate differences between time points within groups. Sphericity was tested using Mauchly’s test, and significance was established at a *p*-value of <0.05.

## 3. Results

### 3.1. LH

**Figure 1:**
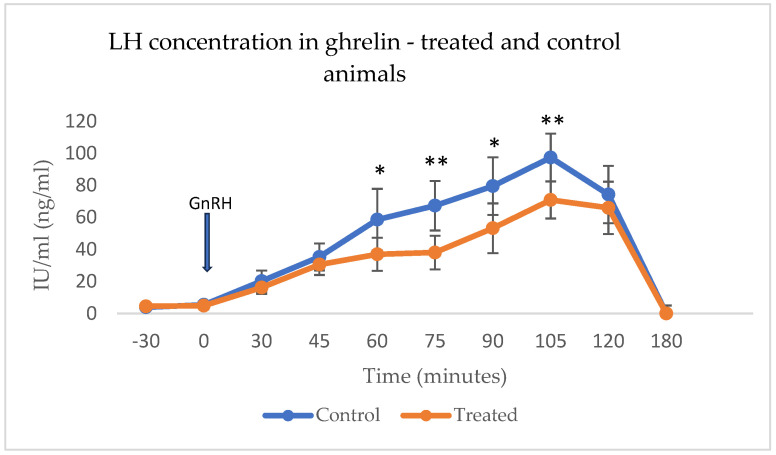
Preovulatory LH surge induced by GnRH administration in synchronized ghrelin-treated (n = 6) and control (n = 6) animals. Asterisks denote significant differences (* *p* < 0.05, ** *p* < 0.01).

The LH concentration did not differ between groups prior to and at the time of GnRH administration. In both groups, 30 min after the GnRH administration LH concentration started to rise until time point 105 and decreased thereafter. The LH levels were steadily higher in control than in treated animals, with significant differences (*p* < 0.03) noted at time points 60, 75, 90, and 105 (Figure 1).

### 3.2. AMH

No significant difference (*p* = 0.8) in AMH concentrations was observed between groups at the time of pump insertion. At the first FSH administration, AMH levels showed a strong tendency (*p* = 0.06) to be higher in control animals compared to treated animals (Control: 3.9 ± 1.2 pg/mL; Treated: 2.1 ± 1.0 pg/mL). At the time of the last FSH injection, hormone concentrations differed significantly between the groups (Control: 4.3 ± 1.0 pg/mL; Treated: 2.0 ± 1.1 pg/mL; *p* = 0.03). In both groups, no significant difference was observed in AMH concentrations between day 0 and the first FSH injection, though the difference in the treated group approached significance (day 0 3.4 ± 1.8 pg/mL, 1st FSH 2.1 ± 1.0 pg/mL, *p* = 0.085).

**Figure 2:**
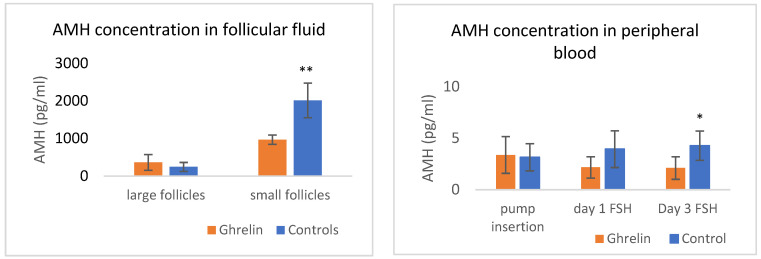
Antimullerian hormone (AMH) concentrations in the follicular fluid of large and small follicles and in the blood at times relative to the day of pump insertion (day 0) and along the 1st and 6th FSH administration in ghrelin-treated and control animals. Follicular fluid AMH concentrations were assayed in 10 animals from each group, while blood AMH levels were determined in 6 animals from each group. Asterisks denote significant differences (* p < 0.05, ** p = 0.0003).

AMH concentrations were significantly higher (*p* < 0.03) in small follicles from control animals (Control: 2.01 ± 0.45 ng/mL; Treated: 0.96 ± 0.45 ng/mL), while no differences (*p* > 0.05) were found in large follicles. AMH concentrations in the peripheral blood and in the follicular fluid are depicted in Figure 2.

### 3.3. Progesterone

**Figure 3:**
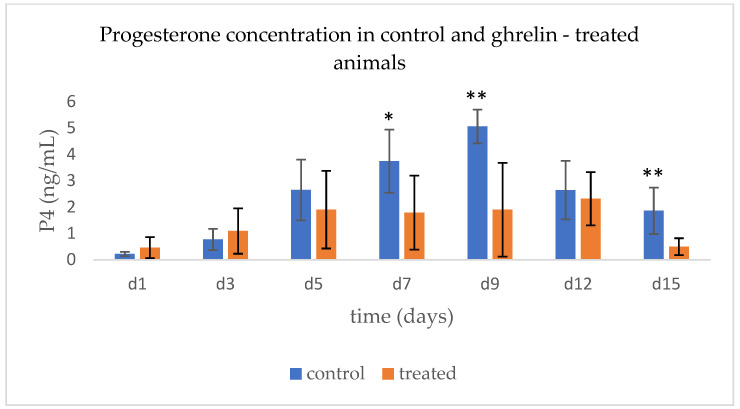
Progesterone concentrations in control (n = 6) and ghrelin-treated animals (n = 6) during the estrus cycle following prostaglandin administration. Asterisks denote significant differences (* *p* < 0.02, ** *p* < 0.006).

As depicted in Figure 3, until day 5, no difference was detected in P4 concentrations between groups. On days 7, 9, and 15, P4 was significantly (*p* < 0.02) higher in the control group. On day 15 in the control group, the progesterone concentration (1.85 ± 1.0 ng/mL) was significantly higher than those of day 1 (0.22 ± 0.1 ng/mL, *p* = 0.006) and day 3 (0.77 ± 0.44 ng/mL, *p* = 0.05), while no differences were detected in the control group.

Following the embryo collection, the duration of the prostaglandin F2α-induced estrous cycle was shorter (*p* = 0.08) in treated animals (17.7 ± 1.0 days) compared to controls (18.4 ± 0.5 days)

### 3.4. Superovulation and Embryo Collection

The superovulatory response, as measured by the number of corpora lutea (CLs) and the embryos collected (morulae and blastocysts), was significantly greater in control ewes compared to those in the treated group. A higher number of small follicles was found in the ovaries of control animals, while the number of large follicles showed no significant difference between the groups. Detailed information regarding the superovulatory response is provided in Table 1.

## 4. Discussion

This study aimed to evaluate the effects of chronic ghrelin infusion on reproductive functions in normally fed sheep. The findings presented provide strong evidence that elevated ghrelin levels negatively impact fertility parameters, affecting the reproductive axis both centrally at the pituitary level and peripherally at the ovarian follicles and corpora lutea.

It is well established that NEB or caloric restriction adversely affects female fertility, influencing all components of the HPO axis (reviews: [1,2,36,37]). Chronic NEB, resulting from prolonged feed restriction in sheep [38], fasting in cattle [39], or increased milk production in high-yielding dairy cows [9], is consistently associated with rising ghrelin concentrations. As described in the above reviews, NEB causes dramatic disturbances in a metabolic and hormonal pathway, inducing or suppressing regulating factors that, in many instances, are interrelated. Given that ghrelin is a factor induced by NEB, we aimed to investigate the effects of exogenously elevated ghrelin levels in animals fed a normal and well-balanced ration.

We opted for DAP-ghrelin over the full-length molecule because its low molecular weight allows for continuous delivery via a mini pump for 28 days. The biological properties of the truncated ghrelin molecule have been previously studied (cattle: [24,25], sheep: [8]) and have been shown to possess similar or even greater potency for stimulating GH secretion compared to the full-length molecule [8,40]. However, Dap-ghrelin cannot be measured using commercially available RIA or ELISA kits; thus, its biological effects can only be indirectly assessed through potential induced responses, such as changes in growth hormone secretion [8,24]; in addition, the effects of DAP-ghrelin on GH secretion was confirmed in our preliminary study.

The results of this study demonstrate that prolonged exposure to elevated ghrelin concentrations leads to a reduction in LH secretion. This finding aligns with observations from other studies across various species, including sheep, cattle, rats, monkeys, and rodents, which have shown that ghrelin priming attenuates LH secretion [8,26,41,42]. In previous research, we found that acylated ghrelin administered peripherally every 15 min on four occasions inhibited the GnRH-induced preovulatory surge of LH and FSH in both cattle and sheep [27,28]. In those studies, the administered dose (1.5 μg/kg of body weight for each injection) of human or bovine acylated ghrelin was significantly higher than the daily dose used in the current experiment (1.25 μg/kg of body weight). Despite this substantial difference in dosage, the resulting suppression of the LH surge was remarkably similar across our studies. This may be due to the higher potency of the truncated molecule [8] or could be related to the different administration methods, where dilution time and the short half-life of ghrelin must be considered.

To our knowledge, this is the first report examining the effects of prolonged ghrelin administration on superovulation and in vivo embryo production in sheep. Our results indicate that ghrelin abolishes the ovarian response to the superovulatory effects of exogenous FSH.

According to early established knowledge, the growth and development of ovarian follicles are governed entirely by FSH [43,44,45,46]. The number of developing follicles is influenced by both the amount and the duration of exposure to high concentrations of FSH [47]. Regardless of follicle size, FSH binds to its receptor (FSH-R) located on granulosa cells, with increased binding in healthy follicles correlating directly with a higher number of receptors and increased granulosa cell numbers [45]. The FSH-R is expressed from the very early stages of follicular development, specifically at the primordial stage, when the follicle consists of two to three layers of granulosa cells and remains active until the follicle fully matures [48].

In our study, although both groups of animals were primed with high doses of exogenous FSH for superovulation, the ghrelin-treated animals did not respond. In fact, the superovulation response in the treated group did not substantially differ from the normal ovulation rate of these crossbred animals. This unexpected outcome is difficult to explain and suggests that attention should be directed toward intrafollicular factors that regulate follicular development or the functionality and quantity of FSH receptors in granulosa cells. In sheep, IGF-1 acts as a significant intrafollicular regulator, acting synergistically with FSH to support follicular development and oocyte maturation [46,49]. In this study, downregulation of IGF-1 is unlikely since we have previously shown that blood IGF-1 concentrations increase in response to ghrelin administration [27]. The inability of ghrelin-treated animals to respond to FSH may be linked to the functionality of the FSH receptor, which is regulated by a complex network involving various kinases such as protein kinases A and C (PKA and PKC), phosphatidylinositol 3-kinase (PI3K), protein kinase B/Akt, p70S6 kinase (p70S6K), and ERK1/2 [50,51]. Previous reports indicate that ghrelin influences Akt1 and ERK1/2 phosphorylation in a time-dependent manner in in vitro matured bovine oocytes and cumulus cells (COCs), affecting their maturation processes [52]. Therefore, it is plausible that ghrelin may similarly affect the granulosa cell population, leading to dysfunction of the FSH receptors. Further, the role of ghrelin as a local regulator in the follicle, mediated by induced dysregulation of centrally produced metabolic pathways such as those of POMC, AgRP, or the NPY, which eventually indirectly affected follicular cells’ functionality, should not be ruled out [15,17,18].

Indirect but compelling evidence of ghrelin’s inhibitory effects on follicular development comes from the analysis of AMH levels in both blood and follicular fluid. At the beginning of the experiment, no differences were observed in peripheral AMH concentrations. AMH is primarily produced by healthy, small developing follicles and is released into the peripheral blood at measurable levels. Thus, blood concentrations of AMH serve as a reliable marker for ovarian reserve in various species, including sheep [53,54,55,56]. Given that both groups had initially similar blood AMH levels, one would expect comparable ovarian responses to FSH; however, this was not observed in our study. The number of small, gonadotropin-dependent follicles and the superovulatory response was lower in the treated group, suggesting that through an unknown mechanism, ghrelin inhibited both spontaneous follicular development (as indicated by AMH levels at the first FSH injection) as well as FSH-induced superovulation. Notably, in treated animals, AMH levels declined within 12 days, i.e., from the time of pump insertion until the first FSH injection, while in control animals, as would be expected, AMH levels remained stable. Furthermore, AMH concentrations in small follicles were higher in the control animals. Our findings regarding control animals align with those of Campbell et al. [56], which showed that small gonadotropin-dependent follicles increase AMH expression following FSH treatment. This being the case, one could assume that ghrelin blunts the FSH-induced overexpression of AMH.

The induced cycle following superovulation was characterized by decreased progesterone secretion and shorter duration in the group of ewes treated with ghrelin. Ewes from both groups exhibited estrus in response to the administered prostaglandin, indicating that all animals developed a healthy and estrogenically active preovulatory follicle. However, it remains unclear whether the presence of ghrelin affected the number of granulosa or luteal cells or their steroidogenic capacity. Regardless, the corpora lutea formed in the treated animals can be classified as inadequate, as they produced low levels of progesterone and had a shorter lifespan than expected. In the treated group, the progesterone concentration on day 15 was similar to those of day 1 and day 3, which belong to the metestrus and early luteal phase, respectively, at which the corpus luteum is not yet fully active. Hence, unlike the control animals, in the treated group, the CL regressed earlier than expected. Our results and the results of others regarding the effects of ghrelin on LH modulation support the hypothesis that the corpora lutea of the treated animals had reduced development and secreting ability due to diminished luteotrophic support. In support of this hypothesis, it has been shown that when ghrelin is introduced into the culture media of human or rabbit granulosa/luteal cells, the synthesis of estradiol, progesterone, and IGF-1 is significantly reduced [57,58,59]; these observations disconnect the effect of LH on the secreting ability of the cells. However, administering high doses of acylated ghrelin for a short period (four doses within one hour) during the early stages of the estrous cycle did not result in changes to progesterone secretion [27]. Overall, these findings suggest that ghrelin has a strong suppressive effect on the secretory function of luteal tissue, with its impact on the steroidogenic capacity of luteal cells being primarily time-dependent rather than dose-dependent. As ghrelin is a strong metabolic signal related to energy status and feed availability, our results highlight the importance of appropriate feeding regimes according to the development or production state of the animals that could eliminate any possible ghrelin-induced adverse effects on fertility parameters.

## 5. Conclusions

We found that prolonged elevated levels of ghrelin in normally fed animals suppress the GnRH-induced LH preovulatory surge, indicating a direct inhibitory effect of ghrelin on the gonadotroph cells of the pituitary. Additionally, our findings regarding the role of ghrelin in follicle development imply a potential disruption of the FSH receptor or the activation of an unknown local pathway, which requires further targeted investigation.

## Figures and Tables

**Table 1:** Mean ovarian response in ghrelin-treated (n = 10) and control ewes (n = 10) after induction of superovulation with 6 decreasing doses of FSH.

	Control	Treated	*p*
No. of CLs	8.3 ± 1.3	2.8 ± 1.3	*p* < 0.001
No. of embryos	5.5 ± 1.8	1.3 ± 0.6	*p* < 0.001
No. of large follicles	1.9 ± 0.8	2.2 + 1.2	*p* > 0.1
No. of small follicles	7.4 ± 1.5	5.5 ± 1.0	*p* < 0.05

*Uterine flushing was performed in 10 control animals; due to low ovarian response, embryo collection was undertaken in 6 treated ewes.*

## Data Availability

The data presented in this study are available upon reasonable request from the corresponding author.
